# Tumor Suppressor 4.1N/*EPB41L1* is Epigenetic Silenced by Promoter Methylation and MiR-454-3p in NSCLC

**DOI:** 10.3389/fgene.2022.805960

**Published:** 2022-06-20

**Authors:** Qin Yang, Lin Zhu, Mao Ye, Bin Zhang, Peihe Zhan, Hui Li, Wen Zou, Jing Liu

**Affiliations:** ^1^ Molecular Biology Research Center and Center for Medical Genetics, School of Life Sciences, Central South University, Changsha, China; ^2^ School of Medical Laboratory, Shao Yang University, Shaoyang, China; ^3^ Molecular Science and Biomedicine Laboratory, College of Biology, College of Chemistry and Chemical Engineering, Collaborative Innovation Center for Chemistry and Molecular Medicine, Hunan University, Changsha, China; ^4^ State Key Laboratory for Chemo/Biosensing and Chemometrics, College of Biology, College of Chemistry and Chemical Engineering, Collaborative Innovation Center for Chemistry and Molecular Medicine, Hunan Univers ity, Changsha, China; ^5^ Department of Histology and Embryology, Xiangya School of Medicine, Central South University, Changsha, China; ^6^ Department of Oncology, The Second Xiangya Hospital of Central South University, Central South University, Changsha, China

**Keywords:** 4.1N/EPB41L1, lung adenocarcinoma (LUAD), methylation, miR-454-3P, non–small-cell lung cancer (NSCLC), lung squamous cell carcinoma (LUSC)

## Abstract

Non–small-cell lung cancer (NSCLC) is divided into three major histological types, namely, lung adenocarcinoma (LUAD), lung squamous cell carcinoma (LUSC), and large-cell lung carcinoma (LCLC). We previously identified that 4.1N/*EPB41L1* acts as a tumor suppressor and is reduced in NSCLC patients. In the current study, we explored the underlying epigenetic mechanisms of 4.1N/*EPB41L1* reduction in NSCLC. The 4.1N/*EPB41L1* gene promoter region was highly methylated in LUAD and LUSC patients. LUAD patients with higher methylation level in the 4.1N/*EPB41L1* gene promoter (TSS1500, cg13399773 or TSS200, cg20993403) had a shorter overall survival time (Log-rank *p* = 0.02 HR = 1.509 or Log-rank *p* = 0.016 HR = 1.509), whereas LUSC patients with higher methylation level in the 4.1N/*EPB41L1* gene promoter (TSS1500 cg13399773, TSS1500 cg07030373 or TSS200 cg20993403) had a longer overall survival time (Log-rank *p* = 0.045 HR = 0.5709, Log-rank *p* = 0.018 HR = 0.68 or Log-rank *p* = 0.014 HR = 0.639, respectively). High methylation of the 4.1N/*EPB41L1* gene promoter appeared to be a relatively early event in LUAD and LUSC. DNA methyltransferase inhibitor 5-Aza-2′-deoxycytidine restored the 4.1N/*EPB41L1* expression at both the mRNA and protein levels. MiR-454-3p was abnormally highly expressed in NSCLC and directly targeted 4.1N/*EPB41L1* mRNA. MiR-454-3p expression was significantly correlated with 4.1N/*EPB41L1* expression in NSCLC patients (r = −0.63, *p* < 0.0001). Therefore, we concluded that promoter hypermethylation of the 4.1N/*EPB41L1* gene and abnormally high expressed miR-454-3p work at different regulation levels but in concert to restrict 4.1N/*EPB41L1* expression in NSCLC. Taken together, this work contributes to elucidate the underlying epigenetic disruptions of 4.1N/*EPB41L1* deficiency in NSCLC.

## Introduction

Lung cancer is the most commonly diagnosed cancer and the most lethal cause of cancer mortality worldwide (Bray et al., 2018). Non–small-cell lung cancer (NSCLC), consisting of lung adenocarcinoma (LUAD), lung squamous cell carcinoma (LUSC), and large-cell lung carcinoma (LCLC) (Rodriguez-Canales et al., 2016), represents major types of lung cancers (80–85%) (D'Addario et al., 2010). Owing to a lack of obvious early symptoms and early-stage diagnosis, most patients with NSCLC are diagnosed in the advanced clinical stage—that is—III or IV (Norouzi and Hardy, 2021). Despite recent advances in NSCLC treatment, less than 15% of the patients eventually survived (Quintanal-Villalonga and Molina-Pinelo, 2019; Norouzi and Hardy, 2021).

Gene promoter methylation and miRNA dysregulation are typical markers of cancer epigenetics (Nebbioso et al., 2018). Gene promoter methylation most commonly occurs at the CpG islands and regulates the gene expression at the transcriptional level (Yang et al., 2014; Zhou et al., 2017; Arechederra et al., 2018). 5–10% of CpG islands in the promoter of genes have been identified as cancer-specifically methylated, which should not be methylated in normal cells (Heller et al., 2013; Olbromski et al., 2020). The methylations of certain genes are of clinical relevance for patients with NSCLC (Heller et al., 2013). MiRNAs are endogenous small non-coding RNAs, which directly bind to the 3′-untranslated regions (3′UTRs) of target mRNAs to regulate the gene expression at the posttranscriptional level. NSCLC patients have widespread dysregulation of miRNA expression (Du et al., 2018; Uddin and Chakraborty, 2018). It has been well-documented that 4.1 family members 4.1N/*EPB41L1* and its homologs (4.1B/*EPB41L3*, 4.1G/*EPB41L2*, and 4.1R/*EPB41*) are lost in various cancers (Yang et al., 2021). However, epigenetic silencing of 4.1 family members in cancers is still largely unknown. Loss of 4.1B/*EPB41L3* is the only case that has been linked to high promoter methylation in cancers (Kikuchi et al., 2005; Zhang et al., 2012). No miRNAs have been found to regulate 4.1 family members.

Our previous studies suggested that 4.1N/*EPB41L1* is abnormally low expressed and exerts anticancer effects in NSCLC (Wang et al., 2016; Yang et al., 2016; Yang et al., 2021). In the current study, for the first time, we focus on identifying the underlying epigenetic disruptions of 4.1N/*EPB41L1* deficiency in NSCLC. We report that promoter hypermethylation and aberrant miR-454-3p expression regulate 4.1N/*EPB41L1* expression at transcriptional and posttranscriptional levels, respectively, but work in concert to restrict its expression in NSCLC.

## Materials and Methods

### Antibodies

Rabbit anti-4.1N antibody was purchased from ATLAS (Bromma, Sweden). Rabbit anti-GAPDH antibody was purchased from Santa Cruz (Santa Cruz Biotechnology, United States).

### Cell Culture

MRC5, 95C, and 95D cells were grown in DMEM medium (Gibco, United States) and supplemented with 10% fetal bovine serum (Gibco, United States). H460 and A549 cells were grown in RPMI 1640 medium (Gibco, United States) and supplemented with 10% fetal bovine serum. All the cells were grown at 37°C in a humidified atmosphere containing 5% CO_2_.

### NSCLC Tissue Samples

Tumor tissues and tumor-adjacent tissues were obtained from the Second Xiangya Hospital of Central South University (Changsha, China). The tissue samples were subjected to qPCR experiments after approval by the Ethics Committee of the Second Xiangya Hospital. Informed consent was obtained from all participating subjects.

### Methylation-Based Analysis

The MethSurv tool (https://biit.cs.ut.ee/methsurv/) (Modhukur et al., 2018) was used to perform the assessment of methylation-based analysis for the 4.1N/*EPB41L1* gene in LUAD and LUSC. The raw data for LUAD and LUSC could be downloaded from the website (https://biit.cs.ut.ee/methsurv/).

### Cell Transfection and Western Blot

MiR-454-3p and the control mimics were purchased from RiboBio (Guangzhou, China) and transfected according to our previously published protocol (Li et al., 2016). Western blot was also performed according to our previous protocol (Yang et al., 2016).

### Targeted Bisulfite Sequencing

The cells were sent to Biomarker Acegene Corporation, Shenzhen, China for targeted bisulfite sequencing (TBS-seq) analysis. 4.1N/*EPB41L1* promoter methylation was assessed according to the previously published method (Gao et al., 2014a; Gao et al., 2014b; Gao et al., 2015; Pan et al., 2018). Methylation levels are defined as the fraction of read counts of ‘C’ in the total read counts of both ‘C’ and ‘T’ for each covered C site. On the basis of such read fraction, methylated cytosine was called using a binomial distribution as in the method described by Lister et al. (2009), whereby a probability mass function is calculated for each methylation context (CpG). Two-tailed Fisherʼs exact test was used to identify cytosines that are differentially methylated between two samples or groups. Only those CGs covered by at least 200 reads in at least one sample were considered for testing.

### 5-Aza-2′-deoxycytidine(5-Aza-CdR)treatment

5-Aza-CdR (Merck, Germany) was diluted in PBS. The cells were seeded in a 6-well plate and treated with 0, 1, or 10 μM 5-Aza-CdR for 48 h. 5-Aza-CdR was replaced every 24 h.

### RNA Extraction and qPCR

Total RNA was isolated using the RNeasy kit (QIAGEN, United States). cDNA was synthesized using the RevertAid H Minus First-Strand cDNA Synthesis Kit (Thermo Scientific, United States). Stem-loop RT primers (RiboBio, China) were used in reverse transcription for miR-454-3p. qPCR was performed using the One-Step qRT-PCR SYBR® Green Kit (Vazyme Biotech, China). The sequences of primers targeting 4.1N/*EPB41L1* and miR-454-3p were used as described earlier (Wang et al., 2010) and designed by Vazyme Biotech (Nanjing, China). U6 small nuclear RNA was used as an internal control for miR-454-3p analysis.

### Dual-Luciferase Reporter Gene Assay

The 3′UTR target sites of 4.1N/*EPB41L1* mRNA were amplified by PCR with genomic DNA from MRC5 cells. The PCR product was cloned in the psiCHECK2 vector (Promega, United States) to construct the wild-type plasmid (psiCHECK2-4.1N-wt). The corresponding mutant psiCHECK2-4.1N-mut was constructed by *in vitro* site-directed mutagenesis (Mut ExpressMultiS Fast Mutagenesis Kit, Vazyme Biotech, China). Bidirectional sequencing was applied to confirm the correct sequence of the two constructs. For the dual-luciferase reporter gene assay, A549 and H460 cells were cultured in a 24-well plate for 24 h and transfected with psiCHECK2-4.1N-wt or psiCHECK2-4.1N-mut plasmids and miR-454-3p mimics or miR-negative-control using the RiboFECT™CP transfection kit (Ribo Biotechnology, China) and Lipofectamine 2000 (Invitrogen, United States). 48 hours after the transfection, the Dual-Luciferase Reporter Assay System (Promega, Madison, WI, United States) was used to measure the luciferase activity according to the manufacturer’s protocol.

### Statistics

All the experiments were performed in triplicate, and statistical analyses were conducted using GraphPad Prism 5.0. The data were presented as the mean ± standard deviation (SD). Student’s *t*-tests were used to calculate the results. A *p*-value < 0.05 was considered significant statistically. DNA methylation values were represented as beta values (range from 0 to 1). Any beta value equal to or greater than 0.6 was considered fully methylated. Any beta value equal to or less than 0.2 was considered to be fully unmethylated. Beta values between 0.2 and 0.6 were considered to be partially methylated. Differential methylation for individual CpG loci was assessed by comparing the beta values. The patients were classified into high-methylation and low-methylation levels based on maxstat (Modhukur et al., 2018). Cox proportional hazards models were used to perform the survival analysis based on methylation levels of the CpG sites. The methylation levels and overall survival time were used as explanatory variables and response variables, respectively, to perform overall survival analysis.

## Results

### Hypermethylation of the 4.1N/*EPB41L1* Gene in NSCLC

**FIGURE 1:**
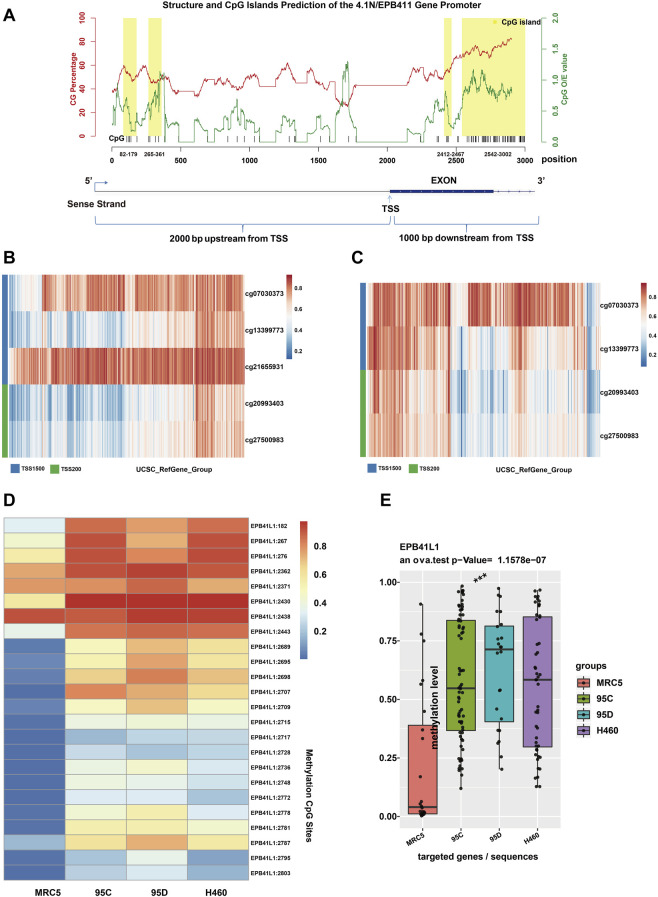
Promoter methylation of the 4.1N/*EPB41L1* gene in NSCLC. **(A)** In the upper panel, CpG island prediction of the 4.1N/*EPB41L1* gene promoter was integrated using the CpGPNP program. The red line represented GC content, the green line represents CpG O/E value, and the yellow box represented four predicted locations of CpG island (range from 82 to 179, 265–361, 2412–2467, and 2542–3002, sequence length >100 bp, GC content >50%, O/E value >0.6). The lower panel represented the structure of the 4.1N/*EPB41L1* gene promoter (range from TSS -2000 to TSS 1000). Coordinate values of abscissas in the lower and upper panels were correlated. TSS -2000 in the lower panel corresponded to 0 in the upper panel. The TSS site in the lower panel corresponded to 2000 in the upper panel. TSS 1000 in the lower panel corresponded to 3000 in the upper panel. **(B,C)** Heatmap depicting the CpG methylation level of the 4.1N/*EPB41L1* gene promoter in LUAD and LUSC patients. Rows and columns represented the CpGs and the patients, respectively. **(D)** Heatmap depicting the methylation levels of the 4.1N/*EPB41L1* gene promoter in normal lung fibroblast cells MRC5 and LCLC cells (95C, 95D, and H460). The row label was the methylation site. The number of row labels corresponded to the coordinate value of abscissa in the upper panel of Panel 1A. Methylation levels were represented as beta values and shown as a continuous variable from blue to red. Any beta value equal to or greater than 0.6 was considered fully methylated. Any beta value equal to or less than 0.2 was considered to be fully unmethylated. Beta values between 0.2 and 0.6 were considered to be partially methylated. **(E)** Boxplots indicating the methylation differences between normal lung fibroblast cells MRC5 and LCLC cells (95C, 95D, and H460). Median methylation levels (show by a thick black line). ***p < 0.001.

**FIGURE 2:**
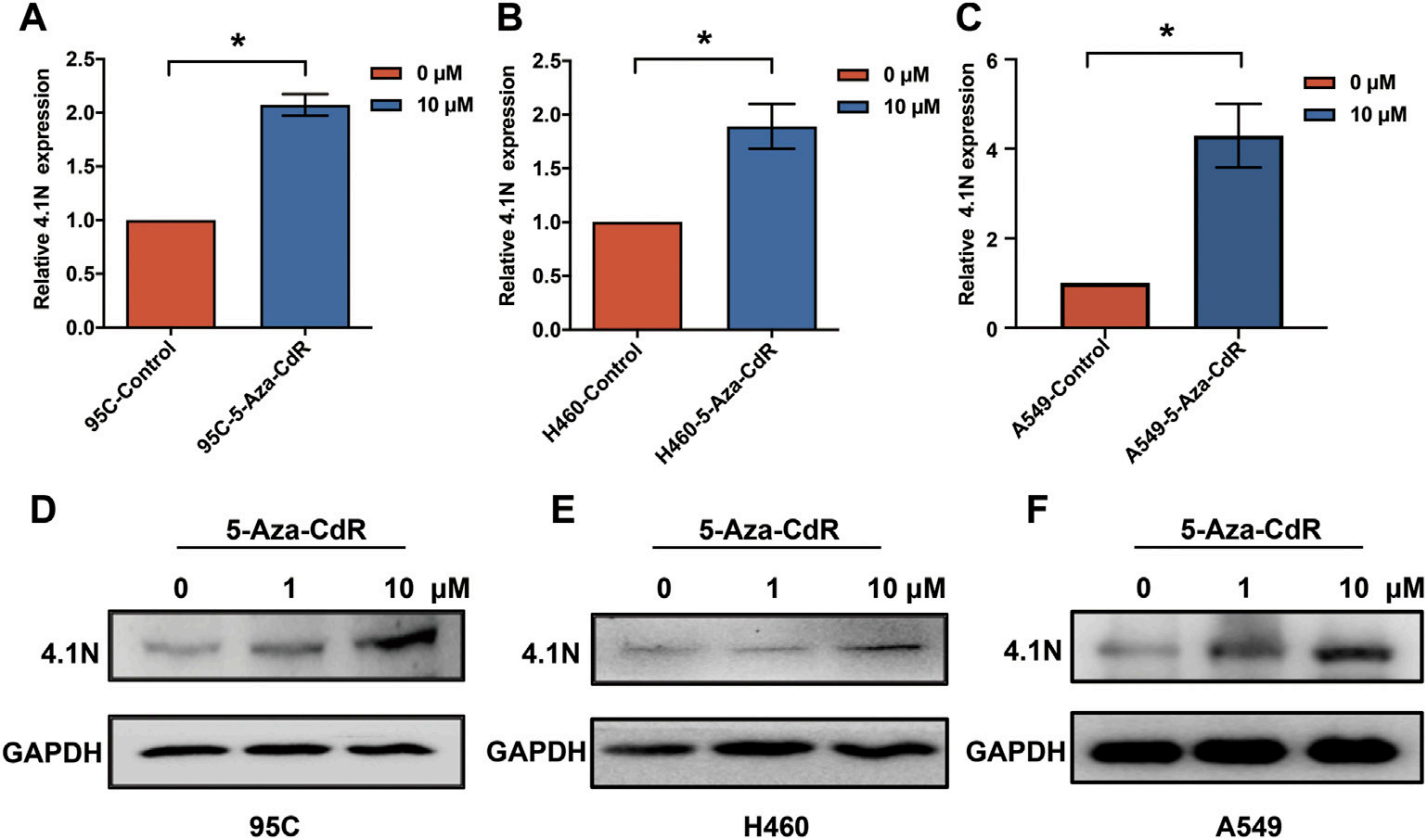
4.1N/*EPB41L1* was restored by methyltransferase inhibitor 5-Aza-CdR in NSCLC cells. **(A–C)** qPCR was performed to evaluate expressions of 4.1N/*EPB41L1* mRNA in 95C, H460, and A549 cells after 0 μM or 1 μM 5-Aza-CdR treatments for 48 h **(D–F)** 95C, H460, and A549 cells were treated with 0, 1 or 10 μM 5-Aza-CdR for 48 h, and then the protein was detected by Western blot. The data were presented as the mean ± SD, *p < 0.05.

Aberrant hypermethylations in the promoter region of genes are considered a major reason for gene silencing in cancer (Lamy et al., 2001). The CpG island methylation prediction using the CpGPNP program (http://forensicdna.kr/cpgpnp/) showed four CpG islands in the 4.1N/*EPB41L1* gene promoter (2,000 bp upstream to 1,000 bp downstream of the transcription start site, Figure 1A). NSCLC predominantly encompasses the LUAD (40% prevalence) and LUSC subtypes (25% prevalence). The MethSurv tool (https://biit.cs.ut.ee/methsurv/) (Modhukur et al., 2018) was used to perform the assessment of methylation-based analysis for the 4.1N/*EPB41L1* gene promoter region (TSS200 and TSS1500) in LUAD and LUSC. The heat map showed that high methylation of the 4.1N/*EPB41L1* gene was prevalent in both LUAD (Figure 1B) and LUSC (Figure 1C). Because the MethSurv tool (https://biit.cs.ut.ee/methsurv/) lacks LCLC data, we investigated the methylation of the 4.1N/*EPB41L1* gene in LCLC cells (95C, 95D, and H460) and normal lung fibroblast cells (MRC5). TBS-seq results showed that promoter methylation of 4.1N/*EPB41L1* was significantly higher in LCLC cells (95C, 95D, and H460) than in normal lung cells (MRC5) (*p* < 0.001) (Figures 1D,E). To further validate the role of methylation in 4.1N/*EPB41L1* gene repression, we treated the LCLC cells (95C and H460) and LUAD cells (A549) with DNA methyltransferase inhibitor 5-Aza-CdR. After demethylation treatment, the 4.1N/*EPB41L1* gene was restored both at mRNA (Figures 2A–C) and protein levels (Figures 2D–F). Taken together, these results indicated that 4.1N*/EPB41L1* gene methylation is a cause of decreased 4.1N*/EPB41L1* expression in NSCLC patients.

### Prognostic Relevance of 4.1N/*EPB41L1* Hypermethylation in LUAD and LUSC

**FIGURE 3:**
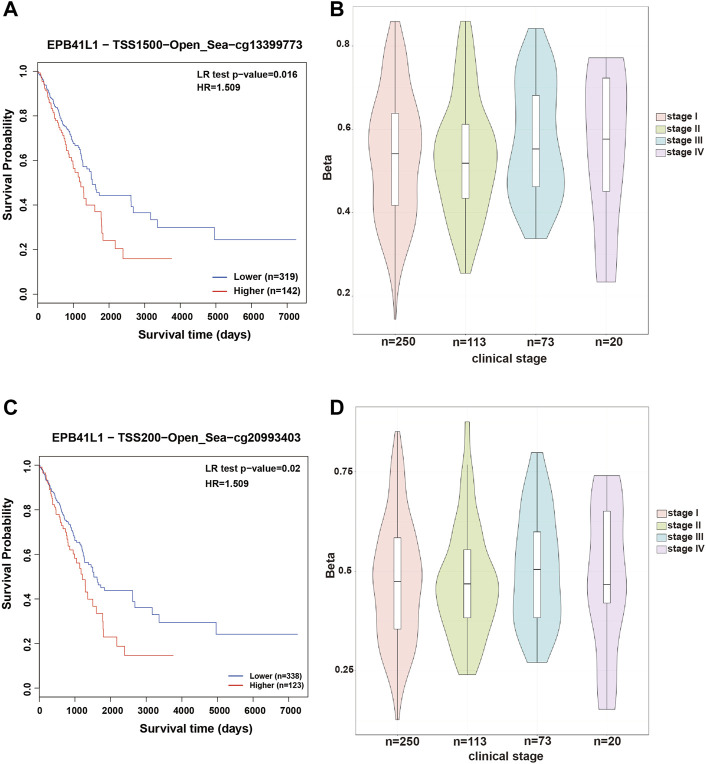
Methylation levels and prognosis of the 4.1N/*EPB41L1* promoter in patients with LUAD. **(A)** Kaplan–Meier curves for cg13399773-4.1N showing survival in lower (beta <0.611, shown in blue) and higher methylation groups (beta *>* 0.611, shown in red) dichotomized by the maxstat method. **(C)** Kaplan–Meier curves for cg20993403-4.1N showing survival in lower (beta <0.59, shown in blue) and higher methylation groups (beta *>* 0.59, shown in red) dichotomized by the maxstat method. **(B,D)** Violin plots showing the methylation levels of cg13399773-4.1N and cg20993403-4.1N among stage I–IV LUAD patients. Median methylation levels (show by a thick black line) and interquartile range were summarized by the boxplot within each violin plot. HR: hazard ratio; LR: log-likelihood ratio; LUAD: lung adenocarcinoma. Methylation levels were represented as beta values. Any beta value equal to or greater than 0.6 was considered fully methylated. Any beta value equal to or less than 0.2 was considered to be fully unmethylated. Beta values between 0.2 and 0.6 were considered to be partially methylated.

**FIGURE 4:**
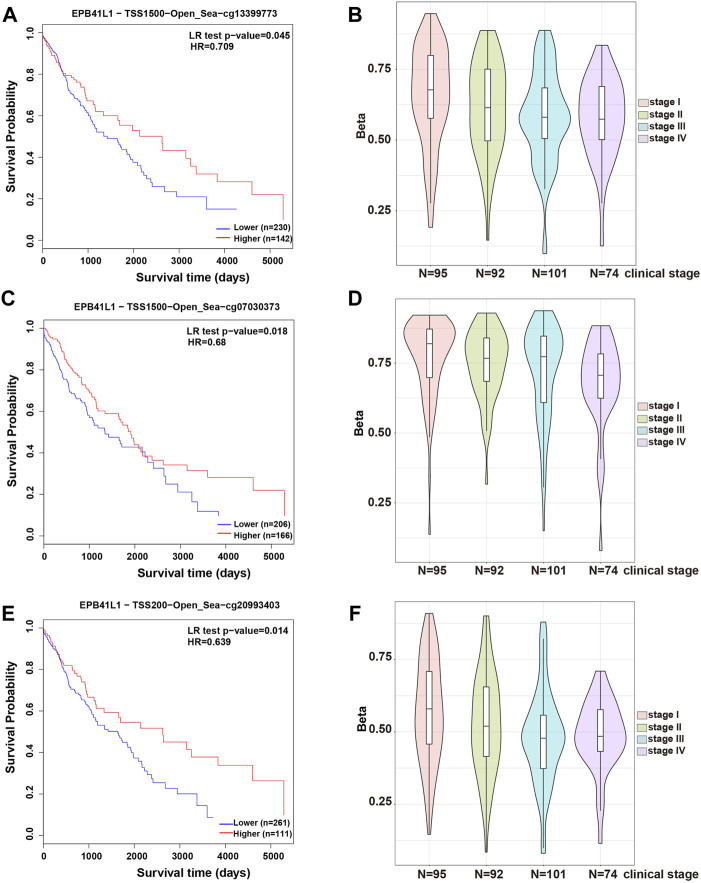
Methylation levels and prognosis of the 4.1N/*EPB41L1* promoter in patients with LUSC. **(A)** Kaplan–Meier curves for cg13399773-4.1N showing survival in lower (beta <0.675, shown in blue) and higher methylation groups (beta *>* 0.675, shown in red) dichotomized by the maxstat method. **(C)** Kaplan–Meier curves for cg07030373-4.1N showing survival in lower (beta <0.796, shown in blue) and higher methylation groups (beta *>* 0.796, shown in red) dichotomized by the maxstat method. **(E)** Kaplan–Meier curves for cg20993403-4.1N showing survival in lower (beta <0.611, shown in blue) and higher methylation groups (beta *>* 0.611, shown in red) dichotomized by the maxstat method. **(B,D,F)** Violin plots showing the methylation levels of cg13399773-4.1N, cg07030373-4.1N, and cg20993403-4.1N among stage I–IV LUAD patients. Median methylation levels (show by a thick black line) and interquartile range were summarized by the boxplot within each violin plot. HR: hazard ratio; LR: log-likelihood ratio; LUSC: lung squamous cell carcinoma. Methylation levels were represented as beta values. Any beta value equal to or greater than 0.6 was considered fully methylated. Any beta value equal to or less than 0.2 was considered to be fully unmethylated. Beta values between 0.2 and 0.6 were considered to be partially methylated.

In most cases, evaluating DNA methylation signature in the promoter region is highly desirable and sensitive for cancer diagnosis and prognosis. The Kaplan–Meier survival curve showed that the higher methylation levels in the promoter (TSS1500, cg13399773 or TSS200, cg20993403) of the 4.1N/*EPB41L1* gene were significantly associated with a shorter overall survival time (Log-rank *p* = 0.02, HR = 1.509 or Log-rank *p* = 0.016, HR = 1.509, respectively) for LUAD patients (Figures 3A,C). Median methylation levels of the two CpG sites (cg13399773 and cg20993403) were high (beta>0.5) at stage I and did not essentially change in tumors of more advanced stages (Figures 3B,D). Unlike the LUAD, the Kaplan–Meier survival curve showed that the higher methylation levels in the promoter (TSS1500 cg13399773, TSS1500 cg07030373, or TSS200 cg20993403) of the 4.1N/*EPB41L1* gene were significantly associated with a shorter overall survival time (Log-rank *p* = 0.045 HR = 0.5709, Log-rank *p* = 0.018 HR = 0.68 or Log-rank *p* = 0.014 HR = 0.639 respectively) for LUSC patients (Figures 4A,C,E). Median methylation levels of the three CpG sites (TSS1500 cg13399773, TSS1500 cg07030373, or TSS200 cg20993403) were high (beta>0.5) from stage I to IV and tended to decline with tumor progression (Figures 4B,D,F). We did not further explore the contrasting prognostic relevance of 4.1N/*EPB41L1* hypermethylation between LUAD and LUSC. However, methylation of the 4.1N/*EPB41L1* gene might be an important mechanism for tumor formation in LUAD and LUSC because high 4.1N/*EPB41L1* gene methylations were observed at stage I. Because the MethSurv tool (https://biit.cs.ut.ee/methsurv/) lacks LCLC data, the prognostic relevance of 4.1N/*EPB41L1* hypermethylation in LCLC was unknown.

### 4.1N/*EPB41L1* mRNA is a Direct Target of miR-454-3p

**FIGURE 5:**
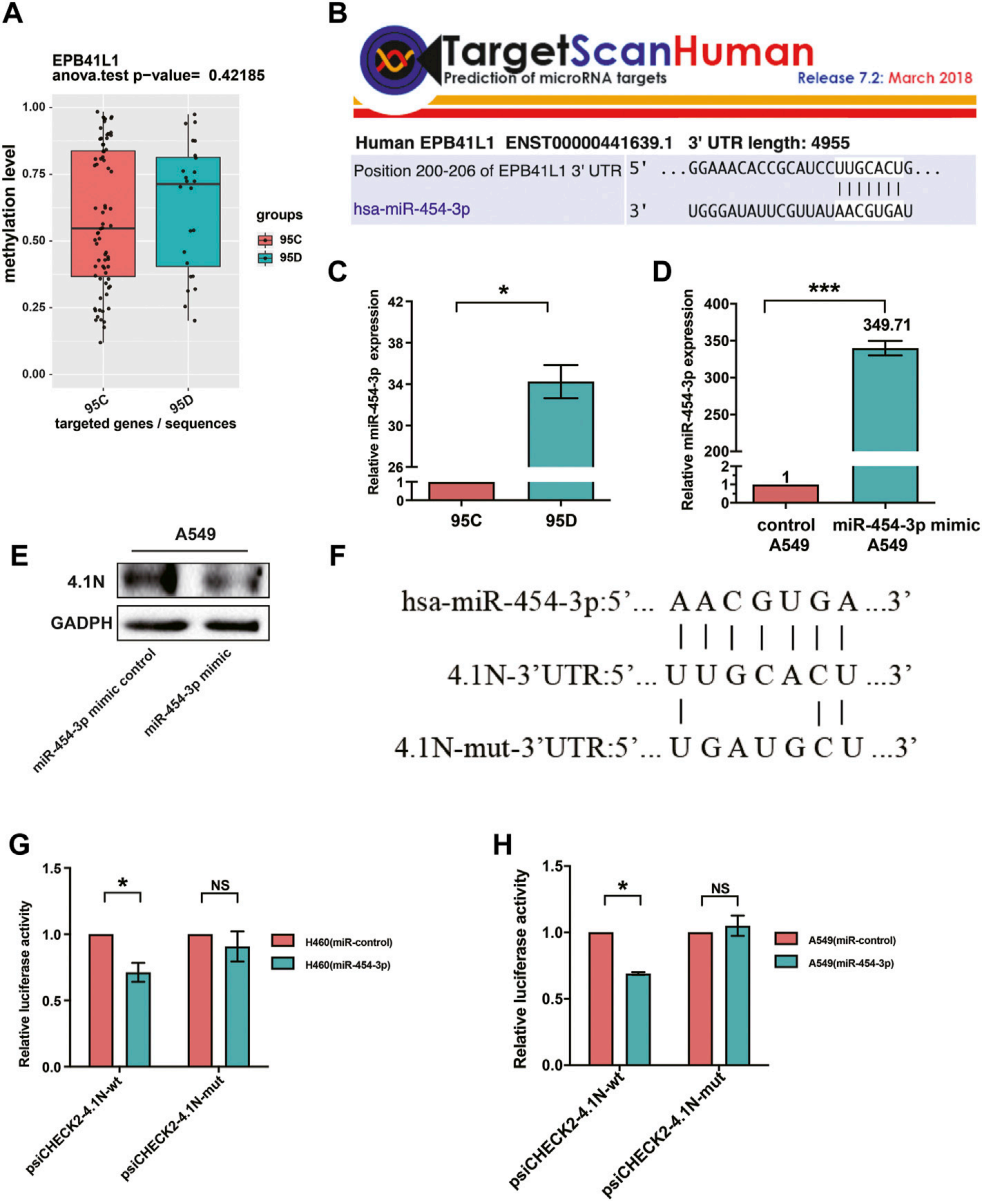
MiR-454-3p directly bound to 3′UTR of 4.1N/*EPB41L1* mRNA. **(A)** There was no significant difference in methylation levels of the 4.1N/*EPB41L1* gene promoter between the two homologous NSCLC subclones 95C and 95D. **(B)** MiR-454-3p was a predicted binding partner of 4.1N/*EPB41L1*. **(C,D)** After transfecting A549 cells with miR-454-3p or negative control mimics, the expression levels of miR-454-3p and protein 4.1N/*EPB41L1* were detected using qPCR and Western blot, respectively. **(E)** miR-454-3p expression was significantly lower in 95C cells than 95D cells. **(F)** Schematic representation of the predicted binding sites of miR-454-3p and 4.1N/*EPB41L1* mRNA and the mutated sequences in potential binding sites. **(G,H)** A549 or H460 cells were co-transfected with miR-454-3p or control mimics and wild-type (psiCHECK2-4.1N-wt) or mutant-type (psiCHECK2-4.1N-mut) plasmids. 48 h later, dual-luciferase activity was measured. The data were presented as the mean ± SD. *p < 0.05 GADPH and U6 were used as loading control of 4.1N/*EPB41L1* mRNA and miR-454-3p, respectively.

We previously described that the 95D cells had remarkably lower 4.1N/*EPB41L1* expression than the 95C cells (Yang et al., 2016). Unexpectedly, although the methylation level of the 95D promoter was higher than that of 95C, there was no significant difference between the two homologous NSCLC subclones 95C/95D (Figure 5A). Over half of all protein-encoding genes are regulated by miRNAs (Turchinovich et al., 2012). Aberrantly high-expressed oncomiRNA silencing cancer suppressing genes are frequently found in NSCLC. Therefore, we investigated the potential miRNAs regulating 4.1N/*EPB41L1* expression. The miRNA-target gene database TargetScan (miRBase:www.mirbase.org) was used to predict the potential miRNAs regulating 4.1N/*EPB41L1*, and miR-454-3p was suggested as a potential miRNA (Figure 5B). qPCR results showed that the miR-454-3p was expressed significantly lower in the 95D cells than in 95C cells (Figure 5C). After overexpressing the miR-454-3p in A549 cells (Figure 5D), the expression level of protein 4.1N/*EPB41L1* was downregulated (Figure 5E).

To further examine if 4.1N*/EPB41L1* was a target gene of miR-454-3p, the dual-luciferase activity assay was applied in A549 and H460 cells. The predicted binding sites of miR-454-3p and 4.1N/*EPB41L1* mRNA and mutant sequences containing four mutated nucleotides are shown in Figure 5F. MiR-454-3p significantly suppressed the luciferase activity, and this suppressive effect was abolished by the mutation in the miR-454-3p-binding region of the 4.1N*/EPB41L1* mRNA 3′UTR in H460 and A549 cells (Figures 5G,H). The abovementioned results signified that abnormally highly expressed miR-454-3p is another epigenetic cause of 4.1N/*EPB41L1* decreasing in NSCLC patients.

### Abnormally High-Expressed miR-454-3p Decreases 4.1N/*EPB41L1* in NSCLC

**FIGURE 6:**
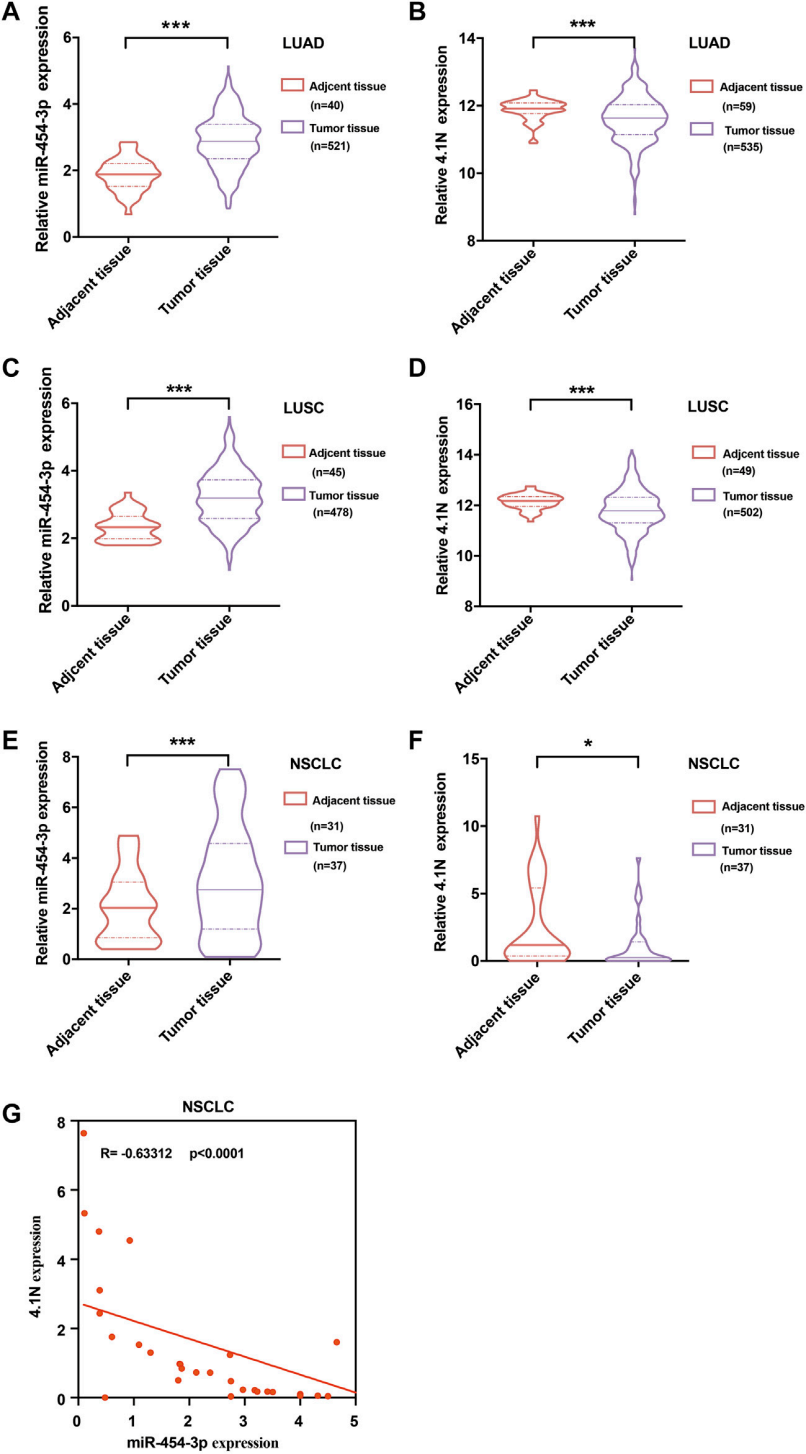
Validation of expression patterns of miR-454-3p and 4.1N/*EPB41L1* mRNA in NSCLC. Relative expressions of miR-454-3p and 4.1N/*EPB41L1* mRNA in LUAD. **(A,B)** and LUSC **(C,D)** were analyzed using the data from the TCGA dataset. **(E-F)** Relative expression of miR-454-3p and 4.1N/*EPB41L1* mRNA in NSCLC and adjacent tissues was measured by qPCR. GADPH and U6 were used as loading control of 4.1N/*EPB41L1* mRNA and miR-454-3p, respectively. **(G)** Correlation between miR-454-3p and 4.1N/*EPB41L1* mRNA was determined using Spearman’s coefficient analysis. The data were presented as the mean ± SD. *p < 0.05 and ***p < 0.001.

TCGA data were used to extract RNA transcript levels of the miR-454-3p. We found that the miR-454-3p was significantly higher in the LUAD (Figure 6A) and LUSC (Figure 6C) tissues in comparison to the adjacent tissues, whereas the 4.1N/*EPB41L1* mRNA was significantly lower in LUAD (Figure 6B) and LUSC (Figure 6D) tissues than the corresponding adjacent tissues. Then, we used qPCR to measure the miR-454-3p and 4.1N/*EPB41L1* mRNA expressions in 37 NSCLC tissues and 31 tumor-adjacent tissues. We found that the expression patterns are consistent with TCGA data (Figures 6E,F). Moreover, the association between miR-454-3p and 4.1N/*EPB41L1* mRNA was validated by Spearman’s coefficient analysis. The MiR-454-3p expression level showed a significantly negative correlation with 4.1N/*EPB41L1* mRNA (r = −0.63, *p* < 0.0001, Figure 6G). Expression patterns of miR-454-3p and 4.1N/*EPB41L1* mRNA showed that abnormally high expression of miR-454-3p negatively regulates 4.1N/*EPB41L1* in NSCLC.

## Discussions

DNA methylation in the promoter region results in gene repression, which is one of the most well-defined epigenetic hallmarks. We previously revealed that downregulated 4.1N/*EPB41L1* exerts antitumor effects by activating the classical Wnt pathway and C-MYC expression in NSCLC (Wang et al., 2016; Yang et al., 2016; Yang et al., 2021). The Wnt pathway disruption driven by methylation of promoter regions plays a key driving role in the high CpG island methylated phenotype LUAD subtype. This subtype is also significantly correlated with the overexpressed *MYC* gene (Cancer Genome Atlas Research, 2014; Duruisseaux and Esteller, 2018). 4.1N/*EPB41L1* has many similar biological characteristics to its homologous 4.1B/*EPB41L3*. High promoter methylation of the 4.1B/*EPB41L3* gene–induced gene-silencing frequently occurs in NSCLC (Kikuchi et al., 2005; Zhang et al., 2012), breast cancer (Heller et al., 2007), renal clear cell carcinoma (Yamada et al., 2006), and prostate cancer (Schulz et al., 2007; Schulz et al., 2010). Thus, we decided to explore the underlying epigenetic disruptions of 4.1N/*EPB41L1* deficiency in NSCLC. Similarly, we found that high 4.1N*/EPB41L1* gene methylation was prevalent in LUAD and LUSC. 4.1B/*EPB41L3* gene methylation is a potential indicator for poor prognosis in NSCLC patients, especially in LUAD patients (Kikuchi et al., 2005). We found that a higher methylation level of 4.1N*/EPB41L1* gene CpG sites (cg13399773 and cg20993403) was potential predictive markers of shorter overall survival in LUAD patients. It is acceptable that patients with higher promoter methylation within the 4.1N*/EPB41L1* gene have a shorter overall survival time because the methylation inhibits tumor suppressor 4.1N*/EPB41L1* expression at the transcriptional level. However, it is a different matter for LUSC, which remains to be elucidated in the future. The 5-year survival rate is less than 15% for NSCLC patients, but the rate can increase to 63% with the early stage of initial diagnosis (van Rens et al., 2000; Geng et al., 2017), thus demonstrating the value of the early diagnosis of NSCLC. 4.1B/*EPB41L3* gene promoter methylation is regarded as an early event in renal clear cell carcinoma (Yamada et al., 2006). In this study, high 4.1N*/EPB41L1* gene methylation was also observed to be a relatively early event in LUAD and LUSC patients, indicating its valuable role in tumorigenesis and potential as an early detection marker.

qPCR results of our sample set and the TCGA database together showed that the miR-454-3p was upregulated in the NSCLC tumor tissue, which contrasted with the results from another independent study (Jin et al., 2019). Interestingly, tumor expression of miR-454-5p in NSCLC is reported as upregulated (Zhu et al., 2016), but another report suggests its expression to be downregulated (Liu et al., 2018). It is not rare when various clinical specimens are evaluated; the expression levels of some miRNAs are different. Because of the complexity of NSCLC development, for these miRNAs, the specimen integration of different NSCLC stages and histological types could impede their expressions together (Zhong et al., 2021). In NSCLC, our study showed aberrantly high-expressed miR-454-3p directly bound to 4.1N/*EPB41L1* mRNA 3′UTR and led to the depression of tumor suppressor 4.1N/*EPB41L1* at the posttranscriptional level, uncovering the known miRNAs regulating 4.1N/*EPB41L1*. It has been reported that the miR-454-3p also acts as oncomiRNA in oral squamous cell carcinoma (Shi et al., 2021), cervical cancer (Guo et al., 2018; Shukla et al., 2019; Song et al., 2019; Li et al., 2021), liver cancer (Li et al., 2019), breast cancer (Ren et al., 2019; Wang et al., 2020), and colon cancer (Li et al., 2018). Moreover, an integrative bioinformatics analysis indicates that miR-454-3p is a biomarker for diagnosing some cancers, including the LUAD (Botling et al., 2013), which needs further confirmation studies in the future.

Epigenetic alterations have been demonstrated to be highly orchestrated from the initiation step to therapy resistance step in lung cancer (Quintanal-Villalonga and Molina-Pinelo, 2019). Although many research studies have revealed the vital anticancer roles of 4.1 family members, the knowledge of the underlying epigenetic mechanism behind their loss in cancers is nearly empty. This study showed that promoter methylation and miR-454-3p were implicated in the expression deregulation of the 4.1N/*EPB41L1* at transcriptional and posttranscriptional levels, respectively. The epigenetic abnormalities are reversible; the application of upregulating 4.1N/*EPB41L1* by targeting DNA methylation and miR-454-3p may represent a promising therapy for NSCLC treatment.

## Data Availability

Publicly available datasets were analyzed in this study. These data can be found here: To investigate the 4.1N/EPB41L1 gene methylation and its relation to NSCLC patient prognosis, TCGA methylation data (https://cancergenome.nih.gov/) of the array features 450k CpG sites covering the 4.1N gene promoter region (TSS200 and TSS1500) were analyzed. The MethSurv tool (https://biit.cs.ut.ee/methsurv/) was used to perform the assessment of methylation-based analysis for the 4.1N/EPB41L1 gene in lung adenocarcinoma (LUAD) and lung squamous cell carcinoma (LUSC). TCGA data for LUAD and LUSC were used to extract RNA transcript levels of the miR-454-3p.

## Abbreviations

3′UTRs, 3′-untranslated regions; 5-Aza-CdR, 5-Aza-2′-deoxycytidine; HR, hazard ratio; LCLC, large-cell lung carcinoma; LR, log-likelihood ratio; LUAD, lung adenocarcinoma; LUSC, lung squamous cell carcinoma; NSCLC, non–small-cell lung cancer; SD, standard deviation; TBS-seq, targeted bisulfite sequencing; TSS, transcription start site.
